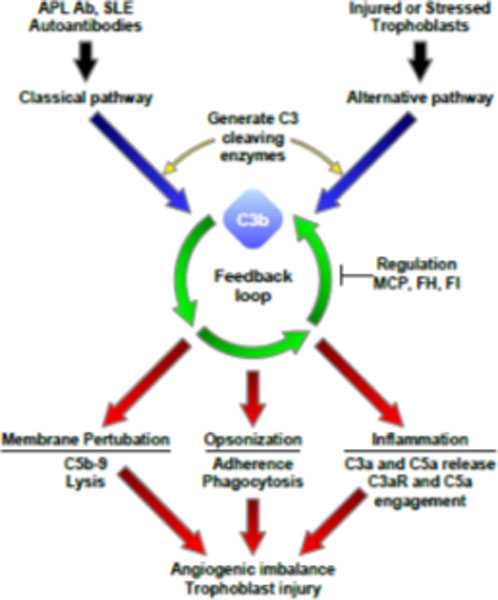# PROMISSE: progress in understanding pregnancy complications in patients with SLE

**DOI:** 10.1186/ar3973

**Published:** 2012-09-27

**Authors:** JE Salmon

**Affiliations:** 1 Autoimmunity and Inflammation Program, Hospital for Special Surgery, New York, NY, USA

## 

Pregnancy complications in women with the antiphospholipid syndrome (APS) and/or SLE include recurrent miscarriage, preeclampsia, placental insufficiency, and intrauterine growth restriction (IUGR). The mechanisms leading to placental and fetal injury *in vivo *are incompletely understood and treatment remains sub-optimal. We have identified complement as an early effector in pregnancy loss and/or IUGR associated with placental inflammation in a mouse model of APS and shown that complement activation drives angiogenic imbalance, placental insufficiency and endothelial injury [1-3] (Figure 1). The PROMISSE Study (Predictors of Pregnancy Outcome: Biomarkers in Antiphospholipid Antibody Syndrome and Systemic Lupus Erythematosus) is a first-time effort to translate our novel findings in mice to humans and determine examine the role of complement as a mediator of complications in patients with antiphospholipid (aPL) antibodies and/or SLE. The following discoveries from PROMISSE will be summarized: lupus anticoagulant is the most powerful predictor of poor pregnancy outcomes in aPL-positive patients [4]; activation of complement early in pregnancy can be detected in the blood of women destined to have preeclampsia; circulating anti-angiogenic factors are biomarkers that predict preeclampsia in patients with SLE and/or aPL antibodies and can be released by products of complement activation; and mutations in complement pathway genes that lead to uncontrolled complement activation are associated with preeclampsia in pregnant patients with SLE and/or aPL antibodies [5]. These findings bring us to closer to identifying those at highest risk for pregnancy complications and intervening to block pathways of injury, such as complement.

**Figure 1:**